# Preferences for life-sustaining treatments in advance decisions: a cross-sectional survey of Taiwanese general public

**DOI:** 10.1186/s12910-025-01242-0

**Published:** 2025-07-04

**Authors:** Daniel Fu-Chang Tsai, Yu-Chen Juang, Chun-Tung Kuo, Ping-Hsueh Lee, Duan-Rung Chen

**Affiliations:** 1 Department & Graduate Institute of Medical Education and Bioethics, College of Medicine, National Taiwan University, Taipei, Taiwan; 2 Institute of Health Policy and Management, College of Public Health, National Taiwan University, Taipei, Taiwan; 3 Institute of Health Behaviors and Community Sciences, College of Public Health, National Taiwan University, Taipei, Taiwan; 4 Center for Survey Research, Research Center for Humanities and Social Sciences, Academia Sinica, Taipei, Taiwan; 5 Department of Geriatrics Medicine, Kuang Tien General Hospital, Taichung, Taiwan; 6 Population Health Research Center, College of Public Health, National Taiwan University, Taipei, Taiwan

**Keywords:** Palliative care, Life support care, Latent class analysis, Advance directive, Advance decision, Advance care planning

## Abstract

**Background:**

Taiwan passed the *Patient Right to Autonomy Act* in 2016 and introduced a legal document called *advance decision* to address dilemmas in making life-sustaining treatment (LST) decisions for incompetent patients. However, the proportion of Taiwanese adults who have completed an *advance decision* remains low, and public preference trends are unclear.

**Methods:**

A cross-sectional telephone survey was conducted among Taiwanese adults using a structured questionnaire to assess preferences regarding five types of LSTs across four hypothetical clinical scenarios (late-stage motor neuron disease, severe dementia, irreversible coma, and terminal cancer). Participants were categorized based on their preference patterns, and factors associated with each subgroup were analyzed.

**Results:**

Of the 3188 individuals contacted, 2440 declined to participate, and 748 (24.3%) respondents were successfully interviewed. A total of 747 responses were included in the analysis. Latent class analysis identified four preference subgroups: pro-forgo (more than half of the respondents), neutral, aggressive, and motor-neuron-disease specific. Older age, higher education, and better quality of life were associated with a greater likelihood of belonging to the pro-forgo group, while being male, unmarried, currently not working, or not residing in northern Taiwan were associated with a lower likelihood.

**Conclusions:**

Most respondents expressed a consistent preference to forgo LSTs in the hypothetical clinical scenarios. This suggests that the *advance decision*, implemented in 2019, may align with public needs. However, given the low completion rate and prevalent preference patterns, policymakers should increase efforts to ensure that those in need have access to appropriate resources and consider implementing a tiered signing process.

**Supplementary Information:**

The online version contains supplementary material available at 10.1186/s12910-025-01242-0.

## Introduction

Delivering goal-concordant care is essential to healthcare quality, but it remains especially challenging for patients at the end of life or those with impaired consciousness [1–3]. Most people prefer to discontinue life-sustaining treatments (LSTs) and opt for palliative or hospice care when their physical condition deteriorates significantly [4–6].However, in the absence of explicit treatment refusals from patients, decisions to terminate or withhold LSTs in those unable to express their wishes can place physicians in ethically complex situations [7, 8]. Although sustaining life is typically viewed as a fundamental benefit, this assumption becomes less clear under such conditions. To address this dilemma, various forms of advance directives (AD), such as living wills, have been developed over the past decades [9]. While the specific content of these documents may vary, they share the common purpose: to allow individuals to articulate their care preferences in advance, based on hypothetical clinical scenarios [10]. When a patient later encounters one of these situations, decisions guided by their AD can be ethically justified under the principle of autonomy [11].

In Taiwan, the legally recognized living will—*the letter of intent for the choice of hospice palliative care or life-sustaining treatment*—was established under the Hospice Palliative Care Act (2000). This document allows individuals to record preferences for “do-not-resuscitate” (DNR) orders and the refusal of LST, which becomes applicable once a terminal condition has been diagnosed by two specialist physicians [12, 13]. However, this document does not apply to non-terminal conditions such as a permanent vegetative state or coma, despite many people’s preference to forgo LST in such circumstances. To address these gaps, Taiwan enacted the *Patient Right to Autonomy Act* in 2016, creating a new legal document known as *advance decision*. This expands the applicable clinical conditions to include irreversible coma, permanent vegetative state, severe dementia, and other incurable and unbearable conditions, such as motor neuron diseases (MNDs), where no effective treatment is available [14].Additionally, the *advance decision* explicitly includes artificial nutrition and hydration (ANH) among the interventions that can be refused in advance. After three years of preparation, this new law and its associated document came into effect in January 2019.

Despite the legal advances, the uptake has been limited. As of December 2023, only about three out of every thousand Taiwanese adults aged 20 or older have signed an *advance decision*. Therefore, public preferences regarding LST in these scenarios remain poorly understood. To assess and reflect on the current state of *advance decision* implementation in Taiwan, we conducted a national telephone survey of the general population. Respondents were presented with four hypothetical clinical scenarios based on the legal conditions defined in the *advance decision* and asked about their preferences regarding five LST options. We then used latent class analysis to identify distinct preference patterns and examined the sociodemographic factors associated with each subgroup.

## Method

### Study design and participants

This cross-sectional, national phone survey was conducted by the Public Opinion Center of Shih Hsin University in November 2019. Participants aged 20 years and older were randomly selected from 22 counties and cities in Taiwan using Chunghwa Telecom landline phone numbers as the sampling frame. Computer-assisted personal interviewing (CAPI) was used to collect and manage data. Verbal consent was obtained after interviewers explained the purpose of the survey and confirmed participants’ age and willingness to participate. In total, 3,188 individuals were contacted, of whom 2,440 refused, yielding 748 completed interviews (response rate: 24.3%). No personally identifiable information was collected.

### Measurement

The Patient Right to Autonomy Act, enforced in Taiwan in January 2019, permits competent adults to document their preferences for LSTs through an advance decision under specific clinical conditions. Four clinical scenarios were designed: irreversible coma, terminal colorectal cancer, severe dementia, and late-stage MNDs. Each scenario involved extreme physical decline and fell within the scope of the Act. Respondents rated their preference for five LSTs—antibiotics for pneumonia, dialysis for renal failure, artificial ventilation, cardiopulmonary resuscitation (CPR), and nasogastric tubes for dysphagia—using a 5-point Likert scale (1 = definitely do not want, 2 = probably do not want, 3 = unsure, 4 = probably want, 5 = definitely want). (See Additional file 1). The questionnaire was adapted from the Life Support Preferences Questionnaire proposed by Coppola [15],which has demonstrated good validaty and has been widely used to assess individual or surrogate preferences for LSTs [16–18].

### Sociodemographic characteristics

The demographic characteristics included gender, age (years), marital status (unmarried, married, divorced/separated/widowed), education level (elementary, junior high, high school, college or above), occupation (white-collar, blue-collar, currently not working, other), and geographic region (northern, central, southern, eastern Taiwan). Other characteristics included self-rated quality of life (1–5 scale), religious affiliation (yes/no), presence of any of the following chronic diseases (hypertension, diabetes, cardiovascular disease, kidney disease, liver disease, cancer, neurological disease, chronic obstructive pulmonary disease; coded as yes/no), and experience caring for a family member with a severe illness or at the end of life (yes/no).

### Data analysis

Descriptive statistics of the LST preferences in hypothetical scenarios are reported. Latent class analysis was conducted to categorize respondents into subgroups according to their preferences for LST in each clinical scenario. The latent class analysis utilizes observed variables to characterize distinct unobserved subgroups (latent classes). We aimed to identify and recognize these unobserved, categorical subgroups. First, we determined the optimal number of latent classes among 2 to 5 groups based on model fit indicators (Akaike information criterion and Bayesian information criterion). We then conducted a one-way ANOVA with Scheffé post-hoc test to compare the differences in attitudes toward the use of LST to determine which groups were the most and least willing to undergo LST. Finally, we defined the group least inclined to undergo LST as the pro-forgo group and used logistic regression to investigate which characteristics were associated with being in this group. We used Stata version 16 to perform latent class analysis. Other data analyses were conducted using the IBM SPSS statistical package (version 25.0, SPSS; Chicago, IL, USA). All statistical analyses were two-tailed.

## Results

### Participants characteristics and general attitudes toward LST

Of the 748 respondents successfully interviewed, 747 were included in the analysis (Table 1); one was excluded due to substantially incomplete data. Among the included participants, 327 were men (43.8%) and 420 were women (56.2%). The mean age was 53.9 years (standard deviation = 14.7 years). Most participants were married (78.8%), and nearly half had a university degree (49.5%). More than 40% were white-collar workers, and more than 40% were not currently working (retired, homemakers, or students). Nearly 80% rated their quality of life as very good or good, whereas only 6.2% felt their quality of life was bad or very bad. Almost one-third had no religious affiliation, and one-third had a chronic illness. More than half had experienced caring for a family member who was seriously ill or at the end of life (54.9%). Table 2 shows that most respondents inclined to refuse (no and probably no) LSTs across all clinical scenarios (70.2–92.8%). Among the five LSTs, artificial ventilation and CPR were the least prefered. Respondents expressed the most definite attitudes in the irreversible coma scenario.

**Table 1:** Participant characteristics (*n* = 747)

Variable	n	%
**Gender**		
Men	327	43.8
Women	420	56.2
**Age** (Mean, SD)	53.9	14.7
**Marital status**		
Unmarried	132	17.7
Married	588	78.8
Divorce/separated/widowed	26	3.5
**Education**		
Elementary school	62	8.3
Junior high school	85	11.4
High school	230	30.8
College or above	369	49.5
**Occupation**		
White collar	306	41.1
Blue collar	85	11.4
Not working	313	42.1
Others	40	5.4
**Quality of life**		
Very good	164	22.3
Good	428	58.2
Fair	99	13.5
Bad	38	5.2
Very bad	7	1.0
**No religion**	238	32.4
**Having chronic disease**	248	33.5
**Having care experience**	407	54.9
**Living area**		
North region	268	35.9
Middle region	160	21.4
South region	177	23.7
East region	142	19.0

**Table 2:** Preferences for five LSTs in four hypothetical clinical conditions (*n* = 747^a^)

	**Irreversible coma**	**Terminal cancer**	**Severe dementia**	**End-stage MNDs**
	**n (%)**	**n (%)**	**n (%)**	**n (%)**
**Antibiotics for severe infection**				
No	578 (77.4)	498 (66.7)	476 (63.7)	366 (49.0)
Probably no	67 (9.0)	73 (9.8)	106 (14.2)	158 (21.2)
Probably yes	67 (9.0)	101 (13.5)	95 (12.7)	121 (16.2)
Yes	31 (4.1)	65 (8.7)	56 (7.5)	75 (10.0)
**Dialysis for renal failure**				
No	587 (78.6)	564 (75.5)	508 (68.0)	411 (55.0)
Probably no	84 (11.2)	78 (10.4)	106 (14.2)	141 (18.9)
Probably yes	47 (6.3)	61 (8.2)	79 (10.6)	106 (14.2)
Yes	24 (3.2)	37 (5.0)	45 (6.0)	63 (8.4)
**Artificial ventilation**				
No	629 (84.2)	601 (80.5)	589 (78.8)	541 (72.4)
Probably no	64 (8.6)	71 (9.5)	82 (11.0)	123 (16.5)
Probably yes	32 (4.3)	40 (5.4)	39 (5.2)	44 (5.9)
Yes	18 (2.4)	30 (4.0)	34 (4.6)	34 (4.6)
**Cardiopulmonary resuscitation**				
No	615 (82.3)	605 (81.0)	581 (77.8)	545 (73.0)
Probably no	59 (7.9)	56 (7.5)	65 (8.7)	77 (10.3)
Probably yes	42 (5.6)	43 (5.8)	48 (6.4)	61 (8.2)
Yes	27 (3.6)	36 (4.8)	47 (6.3)	55 (7.4)
**Nasogastric tube for dysphagia**				
No	588 (78.7)	546 (73.1)	519 (69.5)	454 (60.8)
Probably no	78 (10.4)	72 (9.6)	81 (10.8)	119 (15.9)
Probably yes	48 (6.4)	85 (11.4)	86 (11.5)	106 (14.2)
Yes	25 (3.3)	35 (4.7)	51 (6.8)	53 (7.1)

^a^Missing values (not sure/don't know) below 3.6%

### Latent class analysis and attitudes toward LST

**Fig. 1:**
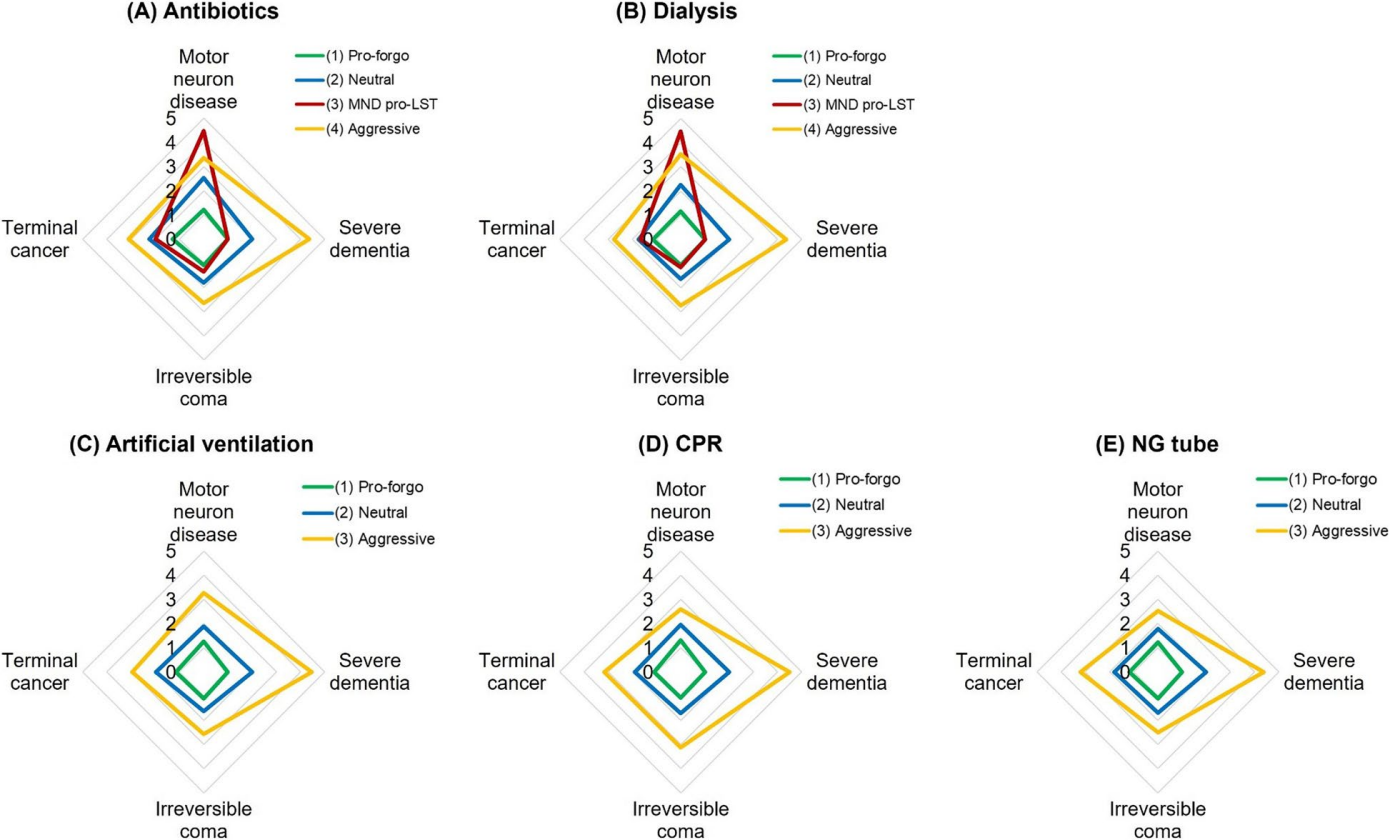
The intention to undergo LST across latent class analysis groups

Based on the model fit indices, the latent class analysis revealed that two treatments, antibiotics and dialysis, fit into four groups (pro-forgo, neutral, MND pro-LST, aggressive). By contrast, artificial ventilation, CPR, and nasogastric tubes fit into three groups (pro-forgo, neutral, aggressive). Table 3 shows the group sizes and the mean scores for willingness to undergo LSTs under the four clinical situations. Higher scores indicate a greater willingness to undergo LST. Among the five LSTs, the pro-forgo group had the highest proportion of participants, ranging from 56.4% to 79.3%. Figure 1 presents the mean willingness scores for each LST in a radar plot. The one-way ANOVA results for the preference for life-sustaining treatment were significant across the groups. According to the Scheffé post-hoc test, the pro-forgo group had the lowest mean scores, indicating the most support for forgoing LST. In contrast, the aggressive group had the highest intention to have LST. And the fourth group was only identified in antibiotics and dialysis, presenting an increased willingness to undergo these two treatments in MND scenarios (Fig. 1).

**Table 3:** Latent class analysis and attitudes toward LST

			Irreversible coma	Terminal cancer	Severe dementia	End-stage MNDs
**Latent class**	**n**	**%**	mean	mean	mean	mean
**Antibiotics**						
(1) Pro-forgo	421	56.4	1.1	1.3	1.0	1.2
(2) Neutral	106	14.2	1.8	2.2	2.0	2.5
(3) MND pro-LST	66	8.8	1.3	2.0	1.0	4.5
(4) Aggressive	154	20.6	2.7	3.1	4.4	3.4
*Post-hoc test*			(4) > (2) > (3) = (1)	(4) > (3) = (2) > (1)	(4) > (2) > (3) = (1)	(3) > (4) > (2) > (1)
**Dialysis**						
(1) Pro-forgo	454	60.8	1.1	1.2	1.0	1.2
(2) Neutral	106	14.2	1.6	1.7	2.0	2.2
(3) MND pro-LST	61	8.2	1.2	1.7	1.0	4.5
(4) Aggressive	126	16.9	2.7	2.7	4.4	3.5
*Post-hoc test*			(4) > (2) > (3) = (1)	(4) > (3) = (2) > (1)	(4) > (2) > (3) = (1)	(3) > (4) > (2) > (1)
**Artificial ventilation**						
(1) Pro-forgo	592	79.3	1.1	1.2	1.0	1.3
(2) Neutral	82	11.0	1.6	2.0	2.0	1.9
(3) Aggressive	73	9.8	2.6	2.9	4.5	3.3
*Post-hoc test*			(3) > (2) > (1)	(3) > (2) > (1)	(3) > (2) > (1)	(3) > (2) > (1)
**CPR**						
(1) Pro-forgo	584	78.2	1.1	1.1	1.0	1.3
(2) Neutral	65	8.7	1.7	1.9	2.0	2.0
(3) Aggressive	98	13.1	3.1	3.2	4.5	3.6
*Post-hoc test*			(3) > (2) > (1)	(3) > (2) > (1)	(3) > (2) > (1)	(3) > (2) > (1)
**NG tube**						
(1) Pro-forgo	525	70.3	1.1	1.2	1.0	1.3
(2) Neutral	81	10.8	1.7	1.8	2.0	2.2
(3) Aggressive	141	18.9	2.5	3.2	4.4	3.7
*Post-hoc test*			(3) > (2) > (1)	(3) > (2) > (1)	(3) > (2) > (1)	(3) > (2) > (1)

### Logistic regression for the pro-forgo group

Table 4 presents the results of the logistic regression model for the pro-forgo group. Overall, men were less willing to forgo LST than women. Specifically, concerning dialysis, artificial ventilation, and CPR, there were 33%, 50%, and 40% fewer odds for men to express a position to withhold treatments than women. Furthermore, older adults were more supportive of forgoing LST. The odds increased by 2% for each additional year of age for antibiotics, dialysis, artificial ventilation, and CPR. Unmarried individuals took a relatively anti-forgo stance than married individuals; 39% to 57% fewer odds of supporting forgoing antibiotics, artificial ventilation, CPR, and nasogastric tubes. Compared to those with elementary education, respondents with higher education had higher odds of supporting forgoing dialysis, artificial ventilation, and CPR. Respondents who did not work had lower odds of supporting forgoing CPR than white-collar workers. Notably, the higher the quality of life of a respondent self-rated, the higher the odds for them to express a positive attitude toward forgoing artificial ventilation, CPR, and nasogastric tube. Additionally, those with experience caring for the family had higher odds of being supportive of forgoing dialysis and CPR. Finally, compared to those living in northern Taiwan, those living in other areas had lower odds of being positive about forgoing artificial ventilation, CPR, or nasogastric tube, especially those living in eastern Taiwan.

**Table 4:** Logistic regression for the pro-forgo group

	**Antibiotics**			**Dialysis**			**Artificial ventilation**			**CPR**			**NG tube**		
	OR	95% CI		OR	95% CI		OR	95% CI		OR	95% CI		OR	95% CI	
**Men**	0.83	(0.59 − 1.16)		**0.67**	**(0.48 − 0.95)**		**0.50**	**(0.33 − 0.75)**		**0.60**	**(0.40 − 0.90)**		0.76	(0.53 − 1.09)	
**Age**	**1.02**	**(1.01 − 1.04)**		**1.02**	**(1.00 − 1.04)**		**1.02**	**(1.00 − 1.04)**		**1.02**	**(1.00 − 1.04)**		1.01	(1.00 − 1.03)	
**Marital status (Married)**															
Unmarried	**0.61**	**(0.37 − 1.00)**		0.63	(0.39 − 1.04)		**0.43**	**(0.24 − 0.76)**		**0.51**	**(0.29 − 0.91)**		**0.46**	**(0.28 − 0.77)**	
Divorced/separated/widowed	1.02	(0.43 − 2.43)		0.90	(0.38 − 2.14)		1.21	(0.38 − 3.85)		3.30	(0.74 − 14.81)		1.22	(0.46 − 3.25)	
**Education (Elementary)**															
Junior high school	1.83	(0.87 − 3.88)		**3.48**	**(1.61 − 7.50)**		**2.54**	**(1.02 − 6.32)**		2.06	(0.88 − 4.81)		1.45	(0.63 − 3.34)	
High school	1.35	(0.71 − 2.55)		**1.95**	**(1.04 − 3.66)**		**2.46**	**(1.15 − 5.28)**		1.99	(0.97 − 4.11)		1.27	(0.62 − 2.60)	
College or above	0.95	(0.49 − 1.84)		1.64	(0.85 − 3.15)		**2.52**	**(1.13 − 5.62)**		**2.35**	**(1.09 − 5.06)**		0.90	(0.43 − 1.88)	
**Occupation (White collar)**															
Blue collar	0.69	(0.40 − 1.20)		0.92	(0.53 − 1.60)		0.77	(0.41 − 1.44)		0.56	(0.30 − 1.03)		0.86	(0.48 − 1.52)	
Not working	0.79	(0.53 − 1.16)		0.73	(0.50 − 1.08)		0.78	(0.48 − 1.27)		**0.53**	**(0.33 − 0.86)**		0.86	(0.57 − 1.31)	
Others	1.35	(0.66 − 2.74)		1.25	(0.61 − 2.56)		0.97	(0.41 − 2.27)		0.76	(0.33 − 1.77)		1.87	(0.82 − 4.25)	
**Quality of life**	1.13	(0.93 − 1.38)		1.07	(0.88 − 1.31)		**1.39**	**(1.10 − 1.76)**		**1.39**	**(1.10 − 1.75)**		**1.38**	**(1.12 − 1.71)**	
**No religion**	1.27	(0.89 − 1.81)		1.27	(0.88 − 1.81)		1.36	(0.86 − 2.13)		1.26	(0.82 − 1.95)		1.22	(0.83 − 1.80)	
**Having chronic disease**	1.16	(0.81 − 1.66)		0.95	(0.66 − 1.37)		1.38	(0.86 − 2.20)		1.14	(0.72 − 1.78)		1.21	(0.81 − 1.81)	
**Having care experience**	1.31	(0.96 − 1.80)		**1.46**	**(1.06 − 2.00)**		1.31	(0.88 − 1.94)		**1.53**	**(1.05 − 2.25)**		1.06	(0.75 − 1.49)	
**Living Area (North)**															
Middle	1.39	(0.90 − 2.16)		0.86	(0.55 − 1.34)		0.66	(0.37 − 1.16)		0.81	(0.47 − 1.40)		**0.57**	**(0.35 − 0.92)**	
South	1.26	(0.83 − 1.91)		0.79	(0.52 − 1.20)		**0.55**	**(0.33 − 0.94)**		0.83	(0.50 − 1.38)		**0.61**	**(0.39 − 0.97)**	
East	1.05	(0.67 − 1.63)		0.91	(0.58 − 1.44)		**0.46**	**(0.26 − 0.82)**		**0.58**	**(0.34** − 0.99)		**0.52**	**(0.32 − 0.86)**	

*OR* Odds ratio, *CPR* Cardiopulmonary resuscitation, *NG tube* Nasogastric tube. Bold font type indicates statistically significant associations at *p* < 0.05

## Discussion

### The latent class of preferences for LSTs

The latent class analysis revealed that certain groups exist in the Taiwanese general public, which was sorted according to their preference patterns regarding LST. For artificial ventilation, CPR, and nasogastric tube, three subgroups were identified (pro-forgo, neutral, and aggressive). For antibiotics and dialysis, four subgroups were identified (pro-forgo, neutral, aggressive, and MND-specific pro-LST). More than half of the respondents belonged to the pro-forgo group, and we identified specific sociodemographic characteristics associated with the pro-forgo group.

The pro-forgo group comprised the majority of the study population (ranging from 56.4% to 79.2%), indicating that most respondents intended to forgo all life-sustaining treatments under the specified clinical conditions. Similar findings have been reported in surveys conducted in other countries [4–6].

The neutral group consisted of individuals who didn’t have a definite preference; their choice may vary depending on the situation (ranging 8.7% ~ 14.2%). Several factors—including treatment burden, expected outcome, and the likelihood of the outcomes— can influence preferences regarding LST [19].The treatment burden is a multidimensional concept encompassing both subjective and objective aspects, such as the invasiveness, impact on health or well-being, feasibility of adherence to the treatment, potential financial cost, and the burden on significant others [20].One Korean study also found that the perception of socioeconomic burden influenced attitudes toward who should make the end-of-life decision—whether the patient or the family [21].Since the scenarios in our questionnaire were limited to outcome-related information (e.g., survival period, with or without functional or cognitive impairment), the variability in preferences observed within the neutral group was likely due to differing individual perceptions of treatment burden.

Participants in the aggressive group (ranging from 9.8% to 20.6%) preferred all active treatments across all scenarios. Notably, within this group, more individuals accepted tube feeding than artificial ventilation (18.9% vs. 9.8%). Similar findings have been reported in Japanese studies [6–23]. This suggests that participants in the aggressive group regarded tube feeding as a more acceptable intervention than artificial respiration. In many cultures, feeding is considered a form of basic care and holds symbolic and social significance [24].As a result, withholding or withdrawing feeding tubes has sparked more controversy than other LSTs [25–28].However, tube feeding is now recognized as a medical therapy that, like other treatments, should not be used regularly [29]. For patients with severe diseases such as late-stage dementia, tube feeding can cause more harm due to the complications and the potential use of physical restraints [25–30]. Nevertheless, unlike other LSTs, tube feeding in cases of terminal illness or persistent vegetative state may still be considered basic care in some cultures and religions [31].

In addition, respondents in the aggressive group were more willing to undergo LST in the context of severe dementia than in other clinical scenarios, indicating that they may not recognize "severe dementia" as an end stage of the disease. This may due to the prognostic uncertainty surrounding dementia. The disease has a unique trajectory characterized by a prolonged course from diagnosis to death, often without acute deterioration. Moreover, the cognitive impairment associated with dementia complicates clinical judgments [32, 33].As a result, accurately predicting prognosis in dementia is challenging. Patients with late-stage dementia are often more likely to receive active treatment than those with terminal cancer [33, 34].

Finally, the MND-specific pro-LST group (ranging from 8.2% to 8.8%) emerged only in the context of antibiotics and dialysis—treatments generally perceived as lower burden. Aside from their strong preference for these two interventions in MND scenario, this group’s overall treatment preferences were similar to those of the pro-forgo group. MND is less prevalent than cancer, dementia, or coma. Patients suffering from these diseases gradually lose physical function until paralyzed completely, yet retain full cognitive function. Given that this state can persist for a prolonged period,, the desire for low-burden treatments to sustain life may be reasonable, as patients can still communicate meaningfully. This group is therefore likely composed of individuals familiar with the nature of MND.

### Sociodemographic characteristics associated with forgoing LST

Being in the pro-forgo group was associated with specific demographic and social characteristics. Individuals who were older, had higher levels of education, and reported better quality of life were more likely to support forgoing life-sustaining treatment in all clinical scenarios. In contrast, being male, unmarried, currently not working, or reside outside northern Taiwan were associated with a lower likelihood to be in the pro-forgo group, suggesting these individuals may prefer receiving at least one form of LST in the given scenarios.

This study found that gender and age were influential factors in preferences for LST, consistent with previous findings. Most studies have reported that older individuals and women are more likely to decline LSTs [4–6, 22, 23, 35, 36]. However, a study conducted in Singapore reported contrary result for women [35]. A Dutch national survey indicated that individuals with higher educational attainment were more likely to refuse CPR and ANH in cases of advanced cancer and dementia [4], aligning with our findings.

Although some Taiwanese studies have indicated that lower socioeconomic status—measured through occupational categories and income—is associated with more aggressive care in terminal cancer patients [37–40], our study found no such association. This discrepancy may be due to the use of health insurance claims data in previous studies, which do not necessarily reflect patients’ preferences. Moreover, Taiwan's National Health Insurance program enables individuals to access affordable health care, potentially reducing the influence of financial concerns on treatment preferences.

Our study found that self-rated quality of life, being unmarried, and currently not working were significant factors. A review article from the United States noted that among racially or ethnically diverse groups, African Americans tend to prefer life support and generally report lower trust in the healthcare system [41].Although further research is required, we suggest that these factors may be link to individual’s trust in the healthcare system and broader society. Respondents who rated their quality of life positively may have more favorable personal experiences with their environment, fostering great trust and social connections. Conversely, those who are unmarried and currently not working may have fewer social interactions, increasing the risk of perceived social isolation (loneliness), which is known to be a risk factor for feeling unsafe and distrusting the environment; consequently, they will behave more defensively and self-protectively [42, 43].

A regional difference was also observed in preference for tube feeding. Respondents living outside of northern Taiwan were more likely to favor nasogastric tube use. Cultural beliefs may play a stronger role in shaping perceptions of tube feeding. In Taiwanese culture, having a sense of food satisfaction concerns whether a person can die without regret; otherwise, the dead may become tragic starving souls [44].In Taiwan, the legitimacy of withholding or withdrawing tube feeding as a form of LST wasn't acknowledged until the enactment of the Patient Right to Autonomy Act. Individuals residing in or near the capital region (northern Taiwan) may be more attuned to recent legal developments, which could explain the observed regional differences. However, we expect this gap to narrow over time as public awareness increases.

### Strengths and limitations

This study has several limitations. First, because the telephone survey included only landline users, the average age of respondents was relatively high (53.9 years), with over 40% being retirees or homemakers. Second, although we provided concise descriptions and explanations for each disease scenario, respondents may still have found it difficult to fully understand the medical conditions. Third, as the study relied on hypothetical scenarios to assess people's preferences, the findings may not fully reflect real-world decisions—despite prior studies suggesting that preferences for LSTs can be consistent, especially among individuals who have expressed a wish to forgo treatments [45–47]. However, whether these hypothetical preferences translate into actual clinical behavior remains uncertain [48].

A major strength of this study is the use of latent class analysis as the primary statistical method. This approach allows for the identification of unobserved subgroups within the data and enables the analysis of associated factors at the group level than the individual level.

## Conclusion

Most people expressed a consistent intention to refuse LSTs in clinical conditions perceived as incurable or associated with extremely low physical or cognitive function. This finding suggests that the *advance decision* established under the Patient Right to Autonomy Act, enacted in 2019, may effectively respond to public needs. However, despite its potential to help individuals avoid unwanted care in the future, the majority of Taiwanese adults haven’t yet signed this legal document. Policymakers should prioritize investigating the barriers to the uptake of *advance decision* and develop strategies to ensure equitable and accessible resources for those who wish to complete them.

Moreover, our analyses identified several distinct preference patterns within the population, indicating that individuals can be categorized into several groups based on their attitudes toward LSTs. For the majority, the preference to forgo LST remained consistent across various clinical conditions and treatment types. This raises the question of whether the current standardized process for making *advance decisions*, which involves a lengthy (60–90 min) session with a physician explaining all five clinical conditions and requiring separate decisions for each, is necessary for everyone. Healthy individuals may primarily need the opportunity to reflect on end-of-life issues and express a general preference regarding LSTs. In contrast, older adults or those with chronic diseases may require more condition-specific information. A tiered approach that tailors the *advance decision* process to individuals at different life stages may present a more efficient and practical solution.

## Supplementary Information

Additional file 1.

## Data Availability

The datasets used and/or analyzed during the current study are available from the corresponding author, Duan-Rung Chen, upon reasonable request (email:duan@ntu.edu.tw).

## Abbreviations

AD: Advance directive
ANH: Artificial nutrition and hydration
CAPI: Computer-assisted personal interviewing
CPR: Cardiopulmonary resuscitation
DNR: Do-not-resuscitate
LST: Life-sustaining treatment
MND: Motor neuron disease
NG: Nasogastric tube

## References

[1] Sanders JJ, Curtis JR, Tulsky JA. Achieving Goal-Concordant Care: A Conceptual Model and Approach to Measuring Serious Illness Communication and Its Impact. J Palliat Med. 2018;21:S17–27. 10.1089/jpm.2017.0459. 29091522 10.1089/jpm.2017.0459 PMC5756461

[2] Sudore RL, Heyland DK, Lum HD, Rietjens JAC, Korfage IJ, Ritchie CS, et al. Outcomes That Define Successful Advance Care Planning: A Delphi Panel Consensus. J Pain Symptom Manage. 2018;55:245-55.e8. 10.1016/j.jpainsymman.2017.08.025. 28865870 10.1016/j.jpainsymman.2017.08.025 PMC5794507

[3] Dy SM, Kiley KB, Ast K, Lupu D, Norton SA, McMillan SC, et al. Measuring what matters: top-ranked quality indicators for hospice and palliative care from the American Academy of Hospice and Palliative Medicine and Hospice and Palliative Nurses Association. J Pain Symptom Manage. 2015;49:773–81. 10.1016/j.jpainsymman.2015.01.012. 25697097 10.1016/j.jpainsymman.2015.01.012

[4] van Wijmen MP, Pasman HRW, Widdershoven GA, Onwuteaka-Philipsen BD. Continuing or forgoing treatment at the end of life? Preferences of the general public and people with an advance directive. J Med Ethics. 2015;41:599–606. 10.1136/medethics-2013-101544. 25182697 10.1136/medethics-2013-101544

[5] Clarke G, Fistein E, Holland A, Barclay M, Theimann P, Barclay S. Preferences for care towards the end of life when decision-making capacity may be impaired: A large scale cross-sectional survey of public attitudes in Great Britain and the United States. PLoS ONE. 2017;12:e0172104. 10.1371/journal.pone.0172104. 28379955 10.1371/journal.pone.0172104 PMC5381758

[6] Hamano J, Hanari K, Tamiya N. End-of-life care preferences of the general public and recommendations of healthcare providers: A nationwide survey in Japan. BMC Palliat Care. 2020;19:38. 10.1186/s12904-020-00546-9. 32209096 10.1186/s12904-020-00546-9 PMC7093951

[7] Braun UK, McCullough LB. Preventing life-sustaining treatment by default. Ann Fam Med. 2011;9:250–6. 10.1370/afm.1227. 21555753 10.1370/afm.1227 PMC3090434

[8] Lambden JP, Chamberlin P, Kozlov E, Lief L, Berlin DA, Pelissier LA, et al. Association of Perceived Futile or Potentially Inappropriate Care With Burnout and Thoughts of Quitting Among Health-Care Providers. Am J Hosp Palliat Care. 2019;36:200–6. 10.1177/1049909118792517. 30079753 10.1177/1049909118792517 PMC6363893

[9] Sabatino CP. The evolution of health care advance planning law and policy. Milbank Q. 2010;88:211–39. 10.1111/j.1468-0009.2010.00596.x. 20579283 10.1111/j.1468-0009.2010.00596.x PMC2980344

[10] House SA, Schoo C, Ogilvie WA. Advance Directives. In: StatPearls [Internet]. Treasure Island (FL): StatPearls Publishing; 2025. https://www.ncbi.nlm.nih.gov/books/NBK459133/. Accessed 17 June 2025. 29083680

[11] Burt RA. The end of autonomy. Hastings Cent Rep. 2005;Spec No:S9-13. 10.1353/hcr.2005.0089 16468249

[12] Hospice Palliative Care Act [Internet]. 2021. https://law.moj.gov.tw/ENG/LawClass/LawAll.aspx?pcode=L0020066. Accessed 17 June 2025.

[13] Cheng SY, Chen CY, Chiu TY. Advances of Hospice Palliative Care in Taiwan. J Hosp Palliat Care. 2016;19:292–5. 10.14475/kjhpc.2016.19.4.292.

[14] Patient Right to Autonomy Act [Internet]. 2021. https://law.moj.gov.tw/ENG/LawClass/LawAll.aspx?pcode=L0020189. Accessed 17 June 2025.

[15] Coppola KM, Bookwala J, Ditto PH, Lockhart LK, Danks JH, Smucker WD. Elderly adults’ preferences for life-sustaining treatments: The role of impairment, prognosis, and pain. Death Stud. 1999;23:617–34. 10.1080/074811899200803. 10915454 10.1080/074811899200803

[16] Barrio-Cantalejo IM, Gómez RMB, Medina MCC, Cabezas MAC, Cruz MEM, Lupiáñez PQ. Validation of the Life-Support Preferences Questionnaire (LSPQ) for its use in Spain. Aten Primaria. 2008;40:345–9. 10.1157/13124127. 18620636 10.1157/13124127 PMC7659845

[17] Froman RD, Owen SV. Validation of the Spanish life support preference questionnaire (LSPQ). J Nurs Scholarsh. 2003;35:33–6. 10.1111/j.1547-5069.2003.00033.x. 12701524 10.1111/j.1547-5069.2003.00033.x

[18] Suen LJW, Lee HH, Morris DL. Life-sustaining treatment: A comparison of the preferences of Taiwanese older adults and their family caregiver. J Nurs Res. 2013;21:261–9. 10.1097/JNR.0000000000000006. 24241275 10.1097/JNR.0000000000000006

[19] Fried TR, Bradley EH, Towle VR, Allore H. Understanding the treatment preferences of seriously ill patients. N Engl J Med. 2002;346:1061–6. 10.1056/NEJMsa012528. 11932474 10.1056/NEJMsa012528

[20] Sav A, King MA, Whitty JA, Kendall E, McMillan SS, Kelly F, et al. Burden of treatment for chronic illness: a concept analysis and review of the literature. Health Expect. 2013;18:312–24. 10.1111/hex.12046. 23363080 10.1111/hex.12046 PMC5060781

[21] Kwon Y, Shin D, Lee J, Heo DS, Hong YS, Kim SY, et al. Impact of perception of socioeconomic burden on advocacy for patient autonomy in end-of-life decision making: a study of societal attitudes. Palliat Med. 2008;23:87–94. 10.1177/0269216308099244. 18996980 10.1177/0269216308099244

[22] Miyata H, Shiraishi H, Kai I. Survey of the general public’s attitudes toward advance directives in Japan: How to respect patients’ preferences. BMC Med Ethics. 2006;7:E11. 10.1186/1472-6939-7-11. 17044943 10.1186/1472-6939-7-11 PMC1634860

[23] Kissane LA, Ikeda B, Akizuki R, Nozaki S, Yoshimura K, Ikegami N. End-of-life preferences of the general public: Results from a Japanese national survey. Health Policy. 2015;119:1472–81. 10.1016/j.healthpol.2015.04.014. 26032907 10.1016/j.healthpol.2015.04.014

[24] Torres-Vigil I, Cohen MZ, de la Rosa A, Cárdenas-Turanzas M, Burbach BE, Tarleton KW, et al. Food or medicine: ethnic variations in perceptions of advanced cancer patients and their caregivers regarding artificial hydration during the last weeks of life. BMJ Support Palliat Care. 2012;2:276–9. 10.1136/bmjspcare-2012-000205. 10.1136/bmjspcare-2012-000205 PMC3962949 24654201

[25] Lo B, Dornbrand L. Guiding the hand that feeds. Caring for the demented elderly. N Engl J Med. 1984;311:402–4. 10.1056/NEJM198408093110611. 6429539 10.1056/NEJM198408093110611

[26] Siegler M, Weisbard AJ. Against the emerging stream. Should fluids and nutritional support be discontinued? Arch Intern Med. 1985;145:129–31. 10.1001/archinte.145.1.129. 3918521 10.1001/archinte.145.1.129

[27] Derr PG. Why food and fluids can never be denied. Hastings Cent Rep. 1986;16:28–30. 10.1002/j.1552-146x.1986.tb02305.x. 10.1002/j.1552-146x.1986.tb02305.x 3082799

[28] Gillick MR. Rethinking the role of tube feeding in patients with advanced dementia. N Engl J Med. 2000;342:206–10. 10.1056/NEJM200001203420312. 10639550 10.1056/NEJM200001203420312

[29] American Geriatrics Society Ethics Committee and Clinical Practice and Models of Care Committee. American Geriatrics Society feeding tubes in advanced dementia position statement. J Am Geriatr Soc. 2014;62:1590–3. 10.1111/jgs.12924. Epub 2014 Jul 17. 25039796 10.1111/jgs.12924

[30] Volkert D, Beck AM, Cederholm T, Cruz-Jentoft A, Goisser S, Hooper L, et al. ESPEN guideline on clinical nutrition and hydration in geriatrics. Clin Nutr. 2019;38:10–47. 10.1016/j.clnu.2018.05.024. 30005900 10.1016/j.clnu.2018.05.024

[31] Druml C, Ballmer PE, Druml W, Oehmichen F, Shenkin A, Singer P, et al. ESPEN guideline on ethical aspects of artificial nutrition and hydration. Clin Nutr. 2016;35:545–56. 10.1016/j.clnu.2016.02.006. 26923519 10.1016/j.clnu.2016.02.006

[32] Murray SA, Kendall M, Boyd K, Sheikh A. Illness trajectories and palliative care. BMJ. 2005;330:1007–11. 10.1136/bmj.330.7498.1007. 15860828 10.1136/bmj.330.7498.1007 PMC557152

[33] Birch D, Draper J. A critical literature review exploring the challenges of delivering effective palliative care to older people with dementia. J Clin Nurs. 2008;17:1144–63. 10.1111/j.1365-2702.2007.02220.x. 18416791 10.1111/j.1365-2702.2007.02220.x

[34] Hsu YH, Chou MY, Chen HM, Chang WC, Chu CS, Wang YC, et al. The trend of aggressive treatments in end-of-life care for older people with dementia after a policy change in Taiwan. J Am Med Dir Assoc. 2020;21:858–63. 10.1016/j.jamda.2020.04.011. 32507531 10.1016/j.jamda.2020.04.011

[35] Tan WS, Bajpai R, Ho AHY, Low CK, Carl J. Retrospective cohort analysis of real-life decisions about end-of-life care preferences in a Southeast Asian country. BMJ Open. 2019;9:e024662. 10.1136/bmjopen-2018-024662. 10.1136/bmjopen-2018-024662 PMC6367977 30782914

[36] Chen DR, Jerng JS, Tsai DFC, Young Y. Gender differences in the intention to withhold life-sustaining treatments involving severe dementia for self and on behalf of parent or spouse. BMC Palliat Care. 2022;21:171. 10.1186/s12904-022-01062-8. 36203170 10.1186/s12904-022-01062-8 PMC9534740

[37] Huang CY, Hung YT, Chang CM, Juang SY, Lee CC. The association between individual income and aggressive end-of-life treatment in older cancer decedents in Taiwan. PLoS ONE. 2015;10:e0116913. 10.1371/journal.pone.0116913. 25585131 10.1371/journal.pone.0116913 PMC4293148

[38] Chang TS, Chang CM, Hsu TW, Lin YS, Lai NS, Su YC, et al. The combined effect of individual and neighborhood socioeconomic status on nasopharyngeal cancer survival. PLoS ONE. 2013;8:e73889. 10.1371/journal.pone.0073889. 24069242 10.1371/journal.pone.0073889 PMC3771923

[39] Chang CM, Wu CC, Yin WY, Juang SY, Yu CH, Lee CC. Low socioeconomic status is associated with more aggressive end-of-life care for working-age terminal cancer patients. Oncologist. 2014;19:1241–8. 10.1634/theoncologist.2014-0152. 25342317 10.1634/theoncologist.2014-0152 PMC4257740

[40] Chang TS, Su YC, Lee CC. Determinants for aggressive end-of-life care for oral cancer patients. Medicine (Baltimore). 2015;94:e460. 10.1097/MD.0000000000000460. 25634186 10.1097/MD.0000000000000460 PMC4602967

[41] Kwak J, Haley WE. Current research findings on end-of-life decision making among racially or ethnically diverse groups. Gerontologist. 2005;45:634–41. 10.1093/geront/45.5.634. 16199398 10.1093/geront/45.5.634

[42] Cacioppo JT, Hawkley LC. Perceived social isolation and cognition. Trends Cogn Sci. 2009;13:447–54. 10.1016/j.tics.2009.06.005. 19726219 10.1016/j.tics.2009.06.005 PMC2752489

[43] Cole SW, Capitanio JP, Chun K, Arevalo JMG, Ma J, Cacioppo JT. Myeloid differentiation architecture of leukocyte transcriptome dynamics in perceived social isolation. Proc Natl Acad Sci U S A. 2015;112:15142–7. 10.1073/pnas.1514249112. 26598672 10.1073/pnas.1514249112 PMC4679065

[44] Chiu TY, Hu WY, Chuang RB, Chen CY. Nutrition and hydration for terminal cancer patients in Taiwan. Support Care Cancer. 2002;10:630–6. 10.1007/s00520-002-0397-5. 12436222 10.1007/s00520-002-0397-5

[45] Gready RM, Ditto PH, Danks JH, Coppola KM, Lockhart LK, Smucker WD. Actual and perceived stability of preferences for life-sustaining treatment. J Clin Ethics. 2000;11:334–46. 11252917

[46] Wittink MN, Morales KH, Meoni LA, Ford DE, Wang NY, Klag MJ, et al. Stability of preferences for end-of-life treatment after 3 years of follow-up: The Johns Hopkins Precursors Study. Arch Intern Med. 2008;168:2125–30. 10.1001/archinte.168.19.2125. 18955642 10.1001/archinte.168.19.2125 PMC2596594

[47] Barrio-Cantalejo IM, Simón-Lorda P, Molina-Ruiz A, Herrera-Ramos F, Martínez-Cruz E, Bailon-Gómez RM, et al. Stability over time in the preferences of older persons for life-sustaining treatment. J Bioeth Inq. 2013;10:103–14. 10.1007/s11673-012-9417-4. 23288442 10.1007/s11673-012-9417-4

[48] Auriemma CL, Nguyen CA, Bronheim R, Kent S, Nadiger S, Pardo D, et al. Stability of end-of-life preferences: a systematic review of the evidence. JAMA Intern Med. 2014;174:1085–92. 10.1001/jamainternmed.2014.1183. 24861560 10.1001/jamainternmed.2014.1183 PMC8243894

