# Supplementary material for: Preferences for life-sustaining treatments in advance decisions: a cross-sectional survey of Taiwanese general public
Source: BMC Med Ethics. 2025 Jul 4;26:83. doi: 10.1186/s12910-025-01242-0 (PMC12231297; doi:10.1186/s12910-025-01242-0)
Supplement: Supplementary file 1 — Additional file 1. [file 12910_2025_1242_MOESM1_ESM.docx]

**Appendix 1: Survey Tool**

**Scenario 1 (Motor neuron disease):** You have a motor neuron disease, like ALS (Amyotrophic Lateral Sclerosis). You gradually lose your strength and rely on others for help with bathing, dressing, and toileting. The disease has no cure. After a few years of developing to the final stage, the disease makes you complete paralysis, unable to speak, swallow, and even breathe. Your thinking and memory are unaffected.

**Scenario 2 (Severe dementia):** You have severe dementia. You cannot remember things that just happened and cannot think clearly. You cannot recognize family members and friends. Incontinence. You have to rely on others. You have no chance of recovery, and the condition will only get worse. You will end up in a wheelchair.

**Scenario 4 (Terminal cancer):** You have terminal colon cancer and can’t be treated. You are tired and weak, requiring some help with household chores. You have pain that requires the constant use of medication. In the opinion of your doctor, you have no chance of recovery. Your doctor estimates that you have about six months to live.

In this case (Scenario 1,2,4), please answer the following questions:

|  | (1) Definitely do not want | (2) Probably do not want | (3) Unsure | (4) Probably want | (5) Definitely want­ |
| --- | --- | --- | --- | --- | --- |
| 1. If you developed a serious infection, like pneumonia, would you want to use antibiotics to treat the infection? | □ | □ | □ | □ | □ |
| 1. If your kidney no longer worked properly (renal failure), would you want to undergo hemodialysis? | □ | □ | □ | □ | □ |
| 1. If you developed respiratory failure, would you want to be intubated and put on a respirator? | □ | □ | □ | □ | □ |
| 1. If your heart stopped beating or you stopped breathing, would you want to receive cardiopulmonary resuscitation? | □ | □ | □ | □ | □ |
| 1. If your condition becomes such that you lose the ability to take in food or water by mouth, would you want artificial feeding and fluids by inserting a nasogastric tube? | □ | □ | □ | □ | □ |

**Scenario 3 (Irreversible coma):** You have suffered a severe stroke and have been in a coma for six weeks. In your doctor's opinion, you have no chance of regaining awareness. You have already inserted a nasogastric tube and put on a ventilator. Although you rely on others for help with feeding, bathing, dressing, and toileting, you may live in this condition for several years.

In this case (Scenario 3), please answer the following questions:

|  | (1) Definitely do not want | (2) Probably do not want | (3) Unsure | (4) Probably want | (5) Definitely want­ |
| --- | --- | --- | --- | --- | --- |
| 1. Would you want to continue to be artificially fed by a nasogastric tube? | □ | □ | □ | □ | □ |
| 1. Would you want to continue intubated or tracheotomy to use a respirator for a long time? | □ | □ | □ | □ | □ |
| 1. If you developed a serious infection, like pneumonia, would you want to use antibiotics to treat the infection? | □ | □ | □ | □ | □ |
| 1. If your kidney no longer worked properly (renal failure), would you want to undergo hemodialysis? | □ | □ | □ | □ | □ |
| 1. If your heart stopped beating or you stopped breathing, would you want to receive cardiopulmonary resuscitation? | □ | □ | □ | □ | □ |
